# Venetoclax Shows Low Therapeutic Activity in BCL2-Positive Relapsed/Refractory Peripheral T-Cell Lymphoma: A Phase 2 Study of the Fondazione Italiana Linfomi

**DOI:** 10.3389/fonc.2021.789891

**Published:** 2021-12-06

**Authors:** Laura Ballotta, Pier Luigi Zinzani, Stefano Pileri, Riccardo Bruna, Monica Tani, Beatrice Casadei, Valentina Tabanelli, Stefano Volpetti, Stefano Luminari, Paolo Corradini, Elisa Lucchini, Maria Chiara Tisi, Michele Merli, Alessandro Re, Marzia Varettoni, Emanuela Anna Pesce, Francesco Zaja

**Affiliations:** ^1^ Dipartimento Clinico di Scienze Mediche, Chirurgiche e della Salute, Università degli Studi di Trieste, Trieste, Italy; ^2^ Struttura Complessa (SC) Ematologia, Azienda Sanitaria Universitaria Giuliano Isontina, Trieste, Italy; ^3^ Istituti di Ricovero e Cura a Carattere Scientifico (IRCSS) Azienda Ospedaliero-Universitaria di Bologna, Istituto di Ematologia “Seragnoli”, Bologna, Italy; ^4^ Dipartimento di Medicina Specialistica, Diagnostica e Sperimentale, Università degli Studi di Bologna, Bologna, Italy; ^5^ Divisione di Emolinfopatologia, Istituto Europeo di Oncologia Istituti di Ricovero e Cura a Carattere Scientifico (IRCSS), Milano, Italy; ^6^ Divisione di Ematologia, Dipartimento di Medicina Traslazionale, Università del Piemonte Orientale e Azienda Ospedaliera Universitaria (AOU) Maggiore della Carità, Novara, Italy; ^7^ Unità Operativa Complessa (UOC) Ematologia, Ospedale Santa Maria delle Croci, Ravenna, Italy; ^8^ Clinica Ematologica, Azienda Sanitaria Universitaria (AOU) Friuli Centrale, Udine, Italy; ^9^ Ematologia, Azienda Unita Sanitaria Locale Istituti di Ricovero e Cura a Carattere Scientifico (IRCSS) Reggio Emilia, Arcispedale Santa Maria Nuova, Reggio Emilia, Italy; ^10^ Dipartimento Chirurgico Medico Odontoiatrico e di Scienze Morfologiche con interesse Trapiantologico Oncologico e di Medicina Rigenerativa (CHIMOMO), Università di Modena e Reggio Emilia, Modena, Italy; ^11^ Struttura Complessa (SC) Ematologia, Fondazione Istituti di Ricovero e Cura a Carattere Scientifico (IRCSS) Istituto Nazionale dei Tumori, Milano, Italy; ^12^ Ematologia e Terapie Cellulari, Ospedale S. Bortolo, Vicenza, Italy; ^13^ Ematologia “Ospedale di Circolo e Fondazione Macchi-Azienda Socio Sanitaria Territoriale (ASST) Sette Laghi”, Varese, Italy; ^14^ Ematologia, Azienda Socio Sanitaria Territoriale (ASST) Spedali Civili di Brescia, Brescia, Italy; ^15^ Divisione di Ematologia, Fondazione Istituti di Ricovero e Cura a Carattere Scientifico (IRCSS) Policlinico San Matteo, Pavia, Italy; ^16^ Fondazione Italiana Linfomi, Modena, Italy

**Keywords:** peripheral T-cell lymphoma, BCL2 protein, venetoclax, BCL2 inhibition, relapsed/refractory

## Abstract

Patients with relapsed/refractory (R/R) peripheral T-cell lymphoma (PTCL) have a poor prognosis, with an expected survival of less than 1 year using standard salvage therapies. Recent advances in our understanding of the biology of PTCL have led to identifying B-Cell Lymphoma 2 (BCL2) protein as a potential therapeutic target. BLC2 inhibitor venetoclax was investigated in a prospective phase II trial in patients with BCL2-positive R/R PTCL after at least one previous standard line of treatment (NCT03552692). Venetoclax given alone at a dosage of 800 mg/day resulted in one complete response (CR) and two stable diseases (SDs) among 17 enrolled patients. The majority of patients (88.2%) interrupted the treatment due to disease progression. No relationship with BCL2 expression was documented. At a median follow-up of 8 months, two patients are currently still on treatment (one CR and one SD). No case of tumor lysis syndrome was registered. Therefore, venetoclax monotherapy shows activity in a minority of patients whose biological characteristics have not yet been identified.

**Clinical Trial Registration:**

www.clinicaltrials.gov (NCT03552692, EudraCT number 2017-004630-29).

## Introduction

Peripheral T-cell lymphomas (PTCLs) are a rare and heterogeneous group of T-cell neoplasms, accounting for 5%–10% of all non-Hodgkin lymphomas in Western countries. PTCL arises from mature post-thymic lymphocytes, and the most represented subtypes are PTCL not otherwise specified (PTCL-NOS), angioimmunoblastic T-cell lymphoma (AITL) and other nodal T-cell lymphomas of T-follicular helper origin (TFH), and anaplastic large cell lymphoma (ALCL) ALK-positive (ALCL-ALK+) and ALK-negative (ALCL-ALK-) (1). With few exceptions, PTCLs share an aggressive clinical behavior and poor prognosis; the response to induction chemotherapy is often inadequate and/or short-term (2).

The standard of care for PTCL is based on anthracycline-containing regimens [cyclophosphamide, doxorubicin, vincristine, prednisolone ± etoposide (CHOP/CHOEP)], resulting in a 5-year overall survival (OS) of only 30%–40% (3). High-dose chemotherapy followed by autologous stem cell transplantation (ASCT) is recommended in younger chemosensitive patients, but many of them do not receive the transplant because of ineffective disease control or, eventually, relapse soon after (4).

Following the promising results of a phase 3 clinical trial, the US Food and Drug Administration (FDA) recently approved the use of brentuximab vedotin (BV) in combination with cyclophosphamide, doxorubicin, and prednisolone (CHP) as first-line treatment for CD30-positive PTCL (5). However, only a minority of patients in this study had PTCL-NOS, AITL, or TFH, and the benefit of the addition of BV was not demonstrated in this small population.

Relapsed or refractory (R/R) PTCLs are characterized by a dismal prognosis, and treatment in this setting is an unmet medical need. There is no standard of care in R/R patients; gemcitabine-based regimens are frequently used in this setting, ensuring an overall response rate (ORR) of 30%–70% and median progression-free survival (PFS) of 4–11 months (6).

Romidepsin, belinostat, and pralatrexate received FDA approval for the treatment of R/R PTCLs, but the ORR with these agents is 30%, with a median PFS of a few months (6–8). More promising results were reported with BV, which showed remarkable activity in ALCLs (9), but no similar activity was registered in other PTCLs (10). Other compounds are under investigation in clinical trials alone or in combination (e.g., lenalidomide, copanlisib, duvelisib, 5-azacitidine) (11).

Recent advances in understanding the biology of PTCLs led to identifying BCL2, Cluster of differentiation 38 (CD38), and Programmed death-1 (PD-1) as potential therapeutic targets. Regarding BCL2, an immunohistochemical analysis conducted within the Fondazione Italiana Linfomi (FIL) indicated that BCL2, an antiapoptotic protein, is overexpressed in 88% of patients with AITL, 80% of patients with PTCL-NOS, 58% of patients with ALK- ALCL, and 31% of patients with ALK+ ALCL (12). Moreover, an inverse correlation between BCL2 expression and the apoptotic rate has been demonstrated in PTCL (13), and BCL2 overexpression in PTCLs seems to correlate with disease progression through interactions with the p53-dependent pathway (14). Thus, BCL2 may represent a potential therapeutic target.

Venetoclax is an anti-BCL2 agent approved in Europe for the treatment of chronic lymphocytic leukemia (CLL) that has shown therapeutic activity in mantle cell lymphoma (MCL) and other hematological malignancies (15–17). We report the results of a study from FIL that evaluated the activity of venetoclax monotherapy in BCL2-positive R/R PTCL.

## Patients and Methods

FIL_VERT is an open-label, multicenter, single-arm phase II trial with a two-stage Simon design that aimed to evaluate the activity and safety of venetoclax as a single agent in patients with BCL2-positive PTCL-NOS, AITL, or TFH who were R/R after at least one previous standard line of treatment.

Twenty-one Italian centers belonging to FIL were involved in the present study. Patients aged ≥18 years with a diagnosis of R/R PTCL-NOS, AITL, or TFH and an Eastern Cooperative Oncology Group (ECOG) performance status ≤2 were enrolled. Relapse biopsy, if available, or otherwise the initial biopsy was centrally revised (Division of Haematopathology, European Institute of Oncology, Milan, Italy) according to the revised fourth edition of the WHO classification of hematopoietic and lymphoid tumors (1). The percentage of BCL2-positive tumor cells was scored as follows, according to Bossard et al. (18): 4, >75%; 3, 50%–75%; 2, 25%–49%; 1, 5%–24%; 0, <5%. For central review and confirmation of the diagnosis, fresh sections were cut from the paraffin block(s) and used for immunohistochemistry to assess the expression of BCL2. Only patients with ≥25% BCL2-positive tumor cells in the relapse or initial biopsy were included in the study. For all inclusion and exclusion criteria, see **Supplement 1**.

All patients provided written informed consent, and the study was conducted in accordance with the Declaration of Helsinki. The study protocol was approved by the ethical committee of each participating site. The trial was registered at www.clinicaltrials.gov (NCT03552692) and given EudraCT number 2017-004630-29.

The primary objective of the study was to evaluate the efficacy of venetoclax in terms of ORR [complete response (CR) and partial response (PR)] using the Revised Response Criteria for Malignant Lymphoma (Lugano Classification) (19), which was evaluated after three treatment cycles. Secondary objectives included assessment of the CR rate, PR rate, stable disease (SD) rate, overall survival (OS), time to response (TTR), PFS, duration of treatment, and safety. Finally, the explorative objective was to assess the possible relationship between response and BLC2 expression.

Thirty-five patients were planned to be enrolled. Given the two-stage design of the study, a stop in recruitment was planned after the enrollment of 18 patients to perform interim efficacy analysis. To proceed to the second stage, the minimum number of patients with an ORR had to be 3/18. Treatment consisted of oral venetoclax (800 mg/day for 28 days/cycle) administered continuously as a single agent until disease progression, unacceptable toxicity, withdrawal of consent, and/or the investigator determined that further therapy is not in the patient’s best interest. To reduce the risk of tumor lysis syndrome (TLS), the dosage of venetoclax was gradually increased in an initial ramp-up phase (**Supplement 2**). Patients were also hospitalized for the first 72 h of treatment, and intravenous hydration and antihyperuricemic drugs were administered.

OS and PFS were estimated using the Kaplan–Meier product-limit-method. Adverse events (AEs) were encoded according to National Cancer Institute (NCI) Common Terminology Criteria for Adverse Events v. 4.03.

## Results

Between May 2018 and November 2019, 22 patients were enrolled and 17 were determined to be eligible; the diagnosis was not centrally confirmed in one patient, BCL2 was <25% in two patients, one patient withdrew consent, and one patient died before starting the treatment. Patient characteristics are summarized in **Table 1**. Two of the 17 patients had received ASCT before venetoclax monotherapy. BCL2 positivity in lymphoma cells was 25%–50% in nine patients (53%), 51%–75% in one patient (5.8%), and >75% in seven patients (41.2%).

**Table 1:** Patient’s characteristics at the time of enrollment.

Patient’s characteristics	Patients (N = 17)
**Age, median (range)**	70 (29–86)
Age ≥60 years, n (%)	11 (64.7)
Age ≥70 years, n (%)	9 (52.9)
**Sex**	
Male, n (%)	10 (58.8)
Female, n (%)	7 (41.2)
**Histology**	
PTCL-NOS, n (%)	13 (76.5)
AITL, n (%)	4 (23.5)
**Ann Arbor stage**	
I, n (%)	0 (0)
II, n (%)	1 (5.9)
III, n (%)	4 (23.5)
IV, n (%)	12 (70.6)
**ECOG performance status**	
0	6 (35.3)
1	6 (35.3)
2	5 (29.4)
**Systemic symptoms**	
Absent, n (%)	14 (82.3)
Present, n (%)	3 (17.7)
**Bone marrow involvement**	
Negative, n (%)	7 (41.2)
Positive, n (%)	10 (58.8)
Bone marrow involvement ≥20%, n (%)	4 (23.5)
TLS risk group*	
Missing data, n (%)	1 (5.9)
Low risk, n (%)	9 (52.9)
Intermediate risk, n (%)	7 (41.2)
**Status of disease**	
Relapse	5 (29.4)
Refractory	12 (70.5)
**Prior therapy, n (%)**	
1	8 (47.1)
2	7 (41.2)
3	1 (5.9)
4+	1 (5.9)
Previous ASCT	2 (11.7)
**Number of cycles, median (min–max)**	2 (1–16)
1, n (%)	3 (17.6)
2, n (%)	6 (35.3)
3, n (%)	5 (29.4)
≥4, n (%)	3 (17.6)

*TLS risk group is defined by lymph node (LN) size and absolute lymphocyte count (ALC) defining low-risk group for LN <5 cm and ALC <25 × 10^9^/L, intermediate-risk group for LN >5 cm and <10 cm or ALC ≥25 × 10^9^/L, and high-risk group for LN >10 cm or ALC ≥25 × 10^9^/L associated with LN ≥5 cm. PTCL NOS, Peripheral T-cell Lymphoma not otherwise specified; AITL, Angioimmunoblastic T-cell Lymphoma; ECOG, Eastern Cooperative Oncology Group; TLS, tumor lysis syndrome.

**Figure 1:**
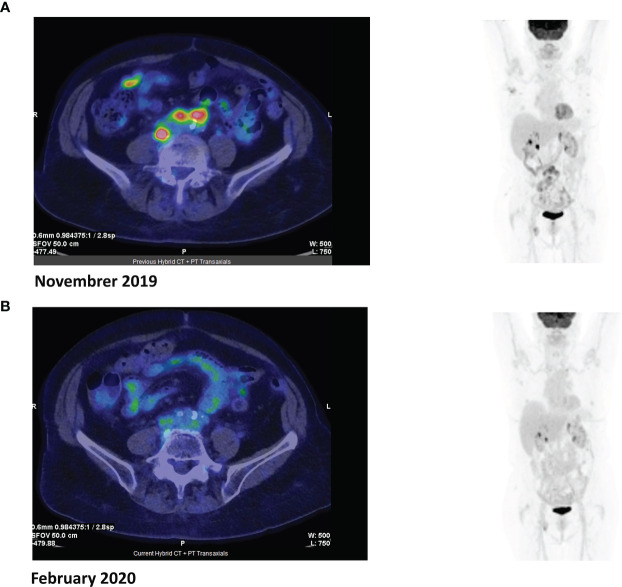
Positron emission tomography (PET) images before **(A)** and after **(B)** three cycles of venetoclax.

Overall therapeutic activity was observed in 3/17 patients (18%). These included one 81-year-old patient in PR after three cycles of treatment with venetoclax (**Figure 1**) that converted to CR at cycle 9 and was maintained 24 months from the beginning of treatment; this patient had PTCL-NOS histology with 50% BCL2 lymphoma cell positivity. He was previously treated with six cycles of age-adjusted CHOP, achieving CR that was maintained for 12 months. Two additional patients had evidence of improvement even without achieving the criteria for PR and for this reason, according to the Lugano classification, were defined as SD. These included a 58-year-old female with relapsed AITL (BCL2 positivity 30%) who received venetoclax for 4 months and experienced disease progression and a 77-year-old male with refractory PTCL-NOS (BCL2 positivity 40%) who is still receiving venetoclax 15 months since the beginning of treatment.

**Figure 2:**
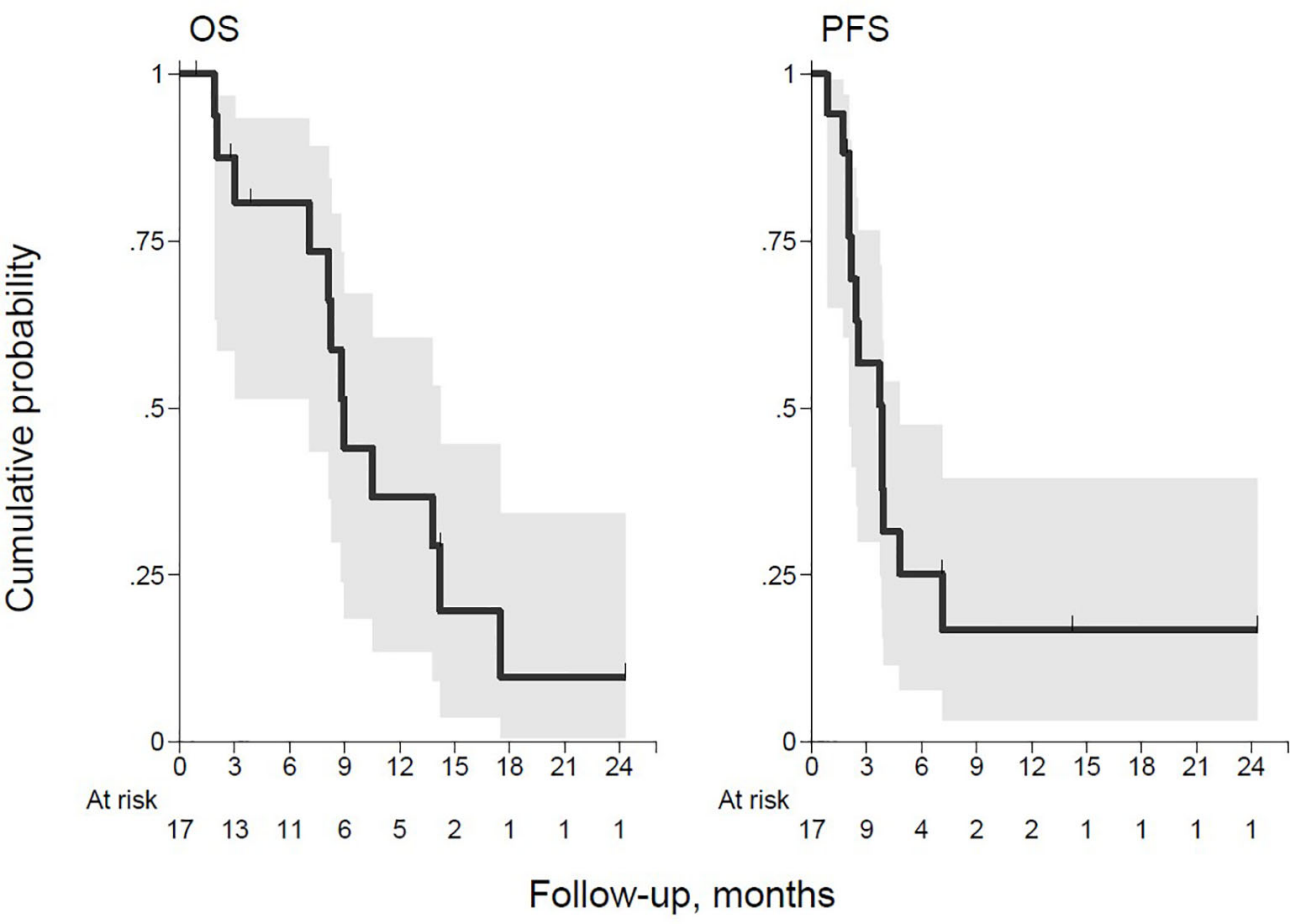
Overall survival (OS) and progression-free survival (PFS).

At a median follow-up of 8 months (range, 1–24 months), two patients are currently still receiving treatment (one CR and one SD). Fifteen patients (88.2%) interrupted the treatment due to disease progression, all but one within five cycles of therapy. Twelve patients (70.6%) died: eight (66.7%) due to Progression of disease (PD) and four (40%) due to worsening of clinical condition and infectious complications. Median OS and PFS were 9 months (95% CI, 7.1–14.2) and 3.8 months (95% CI 2.0–4.8), respectively (**Figure 2**).

A total of 12 patients experienced grade 3–4 hematological toxicities: neutropenia (42%), thrombocytopenia (25%), and anemia (25%). Nine patients (52.9%) experienced grade 3 extrahematological toxicities (two metabolism and nutrition disorders, particularly hyponatremia and hypocalcemia, three cases of pneumonia, one asthenia, two general disorders, and one acute renal failure), whereas no cases of TLS were registered.

Because of the ORR rate <30%, enrollment was stopped at the 17 patients.

## Discussion

FIL_VERT was designed as a proof-of-concept study to evaluate the activity of venetoclax monotherapy in the setting of R/R BCL2-positive PTCLs with the aim of performing a subsequent trial with venetoclax in combination with other agents in case of positive results. Considering that this was the first therapeutic experience with the use of venetoclax in PTCL, the study was designed in two stages according to Simon.

The characteristics of the 17 patients enrolled in the study indicated high risk/poor prognosis. Unfortunately, despite the biologic rationale and efficacy demonstrated by BCL2 inhibition in other hematological malignancies, our preliminary experience showed that venetoclax monotherapy had therapeutic activity in only 18% of patients, with one case of CR. For this reason, the study was stopped after enrollment of the first 17 patients. It is important to point out that two patients (one CR and one SD) experienced a durable therapeutic effect and are still receiving venetoclax 17 and 18 months after beginning treatment, respectively. No apparent relationship could be found, in our small survey, between the level of BCL2 expression and response. The safety profile was in line with previous studies of other hematological malignancies, and no TLS was observed.

The lack of observed response could be due to cellular mechanisms of resistance that have already been observed in other hematological and solid neoplasms. The major determinants of resistance to venetoclax in MCL and CLL patients have been identified as the overexpression and *de novo* synthesis of BCL-X_L_ and MCL-1, antiapoptotic proteins belonging to the BCL2 family (20). Other mechanisms of resistance observed in CLL patients treated with venetoclax included early outgrowth of clones with complex karyotype, mutations in BTG1, aberrations of CDKN2A/B (21), and BCL2 mutation Gly101Val alone or associated with other additional acquired BCL2 resistance mutations, as recently reported (22, 23).

A possible strategy for circumventing resistance could be the association with other agents (chemotherapy and/or monoclonal antibodies or biological agents), as reported below.

In R/R follicular lymphoma (FL), despite a high level of BCL2 expression, venetoclax monotherapy has shown limited efficacy, with an ORR of 38% and only 14% CR rate (17). Several trials are ongoing that combine venetoclax with other molecules and/or chemotherapy in both previously untreated patients and in the setting of R/R FL (e.g., NCT04722601, NCT03980171, NCT02956382).

Increased BCL2 levels are also reported in acute myeloid leukemia (AML) patients, and a majority of AML blasts depend on BCL2 for survival. Furthermore, high expression of BCL2 is associated with poor prognosis with an inferior response to chemotherapy (24). Though single-agent venetoclax has had modest activity in AML, the association with azacitidine in previously untreated patients has shown a synergistic effect, with an incidence of a CR and a CR with incomplete hematological recovery of 71% and a median response duration of 21.1 months (25).

Similarly, in the 15%–20% of R/R multiple myeloma patients who carry chromosomal translocation t(11;14), venetoclax has demonstrated promising activity, with an ORR of 40% and 27%, achieving at least a very good partial response (VGPR) (26). To increase the rate of response, several trials of venetoclax in combination with other agents are ongoing and some preliminary results are available (e.g., venetoclax in association with dexamethasone plus/minus bortezomib) (27).

In conclusion, our data suggest that a small number of R/R patients with PTCL may benefit from venetoclax monotherapy. Similarly to what has been observed in other hematological malignancies, it is possible that the combination of venetoclax plus other cytotoxic or biological agents may translate into a synergistic activity and a higher response rate. BCL2 expression alone seems to not be predictive of the response. Further studies should attempt to identify the mechanisms of response and resistance in this setting and define the predictive markers of venetoclax efficacy.

## Data Availability Statement

The raw data supporting the conclusions of this article will be made available by the authors without undue reservation.

## Ethics Statement

The study protocol was approved by the ethics committee of each participating site. The trial was registered at www.clinicaltrials.gov, NCT03552692, and given the EudraCT number 2017-004630-29. The patients/participants provided their written informed consent to participate in this study.

## Author Contributions

FZ designed the study. FZ and LB wrote the article. All other authors contributed to data collection and data analysis, critically revised the article, and approved the final version of the paper.

## Funding

The study was supported by Abbvie. The funder was not involved in the study design, collection, analysis, interpretation of data, the writing of this article or the decision to submit it for publication.

## Conflict of Interest

PZ has received advisory board fees from Secura Bio, Celltrion, Gilead, Janssen-Cilag, BMS, Servier, Sandoz, MSD, TG Therapeutics, Takeda, Roche, Eusapharma, Kyowa Kirin, Novartis, ADC Therapeutics, Incyte, and Beigene; received consultant fees from MSD, Eusapharma, and Novartis; and received speaker’s bureau fees from Celltrion, Gilead, Janssen-Cilag, BMS, Servier, MSD, TG Therapeutics, Takeda, Roche, Eusapharma, Kyowa Kirin, Novartis, Incyte, and Beigene. SL has received advisory board fees from Roche, BMS/Celgene, Gilead/kite, Jannsen, Genmab, and Regeneron. MV has received advisory board fees from Janssen, Beigene, and Roche and travel expenses from Abbvie. FZ has received advisory board fees from Roche, Celgene, Janssen, Sandoz, Gilead, Novartis, Abbvie, Amgen, Sobi, Argenx, and Grifols and received honoraria for giving lectures to medical meetings from Celgene, Janssen, Gilead, Novartis, Roche, Amgen, Abbvie, and Grifols.

The remaining authors declare that the research was conducted in the absence of any commercial or financial relationships that could be construed as a potential conflict of interest.

## Publisher’s Note

All claims expressed in this article are solely those of the authors and do not necessarily represent those of their affiliated organizations, or those of the publisher, the editors and the reviewers. Any product that may be evaluated in this article, or claim that may be made by its manufacturer, is not guaranteed or endorsed by the publisher.
