# Supplementary material for: Venetoclax Shows Low Therapeutic Activity in BCL2-Positive Relapsed/Refractory Peripheral T-Cell Lymphoma: A Phase 2 Study of the Fondazione Italiana Linfomi
Source: Front Oncol. 2021 Dec 6;11:789891. doi: 10.3389/fonc.2021.789891 (PMC8685372; doi:10.3389/fonc.2021.789891)
Supplement: Supplementary file 1 [file DataSheet_1.docx]

Supplementary Material

**Supplemental 1**

**Inclusion Criteria**

- Histologically documented diagnosis of BCL-2 positive PTCL-NOS, AITL, TFH as defined in the 2016 edi-tion of the World Health Organization (WHO) classification. Only patients with percentage of BCL-2 posi-tive tumor cells ≥ 25% in the relapse biopsy, if available, or otherwise in the initial biopsy, will be included into the study Age ≥ 18 years
- Relapsed or refractory to at least one previous standard line of treatment
- Eastern Cooperative Oncology Group (ECOG) performance status (PS) ≤ 2
- At least one site of measurable nodal or extranodal disease at baseline ≥ 2.0 cm in the longest transverse diameter as determined by CT scan (MRI is allowed only if CT scan cannot be performed). Note: Patients with only bone marrow involvement are eligible
- Adequate hematological counts defined as follows:
- Absolute Neutrophil count (ANC) > 1.0 x 109/L unless due to bone marrow involvement by lymphoma
- Platelet count ≥ 50.000/mm3 unless due to bone marrow involvement by lymphoma
- Adequate renal function defined as follows:
- Creatinine clearance ≥ 30 mL/min
- Adequate hepatic function per local laboratory reference range as follows:
- Aspartate transaminase (AST) and alanine transaminase (ALT) ≤ 3.0 x ULN
- Bilirubin ≤1.5 x ULN (unless bilirubin rise is due to Gilbert’s syndrome or of non-hepatic origin)
- Subject understands and voluntarily signs an informed consent form approved by an Independent Ethics Committee (IEC)/Institutional Review Board (IRB), prior to the initiation of any screening or study-specific procedures
- Subject must be able to adhere to the study visit schedule and other protocol requirements
- Subject must be able to swallow capsules or tablets
- Life expectancy ≥ 3 months
- Women must be:
- postmenopausal for at least 1 year (must not have had a natural menses for at least 12 months)
- surgically sterile (have had a hysterectomy or bilateral oophorectomy, tubal ligation, or otherwise be in-capable of pregnancy),
- completely abstinent (periodic abstinence from intercourse is not permitted) or if sexually active, be practicing a highly effective method of birth control (e.g., prescription oral contraceptives, contracep-tive injections, contraceptive patch, intrauterine device), double barrier method (e.g.: condoms, dia-phragm, or cervical cap, with spermicidal foam, cream, or gel, male partner sterilization) as local regu-lations permit, before entry, and must agree to continue to use the same method of contraception throughout the study. They must also be prepared to continue birth control measures for at least 1 month after terminating treatment
- Women of childbearing potential must have a negative pregnancy test at screening
- Men must agree to use an acceptable method of contraception (fort themselves or female partners as listed above) for the duration of the study. Men must agree to use a double barrier method of birth control and to not donate sperm during the study and for 1 month after receiving the last dose of study drug
- Male even if surgically sterilized (i.e., status postvasectomy) must agree to 1 of the following:
- practice effective barrier contraception during the entire study treatment period and through 1 months after the last dose of study drug, or
- agree to practice true abstinence, when this is in line with the preferred and usual lifestyle of the sub-ject. (Periodic abstinence [e.g., calendar, ovulation, symptothermal, postovulation methods for the fe-male partner] and withdrawal are not acceptable methods of contraception.)

**Exclusion criteria**

- Histological diagnosis different from BCL-2 positive PTCL-NOS, AITL, and TFH
- Allogeneic or autologous stem cell transplant within 6 months prior to the informed consent signature
- Treatment with any of the following within 7 days prior to the first dose of study drug:
- steroid therapy for anti-neoplastic intent
- moderate or strong cytochrome P450 3A (CYP3A) inhibitors
- moderate or strong CYP3A inducers
- Subject has received any anti-cancer therapy including chemotherapy, immunotherapy, radiotherapy, in-vestigational therapy, including targeted small molecule agents within 14 days prior to the first dose of study drug (Note: if clinically indicated a single administration of Vincristine 1 mg before initiating treat-ment is admitted).
- History of CNS involvement by lymphoma
- Administration or consumption of any of the following within 3 days prior to the first dose of study drug:
- grapefruit or grapefruit products
- Seville oranges (including marmalade containing Seville oranges)
- star fruit
- Previous treatment with a BCL-2 family protein inhibitor
- Subject is known to be positive for HIV (HIV testing is not required)
- Cardiovascular disease (NYHA class ≥2)
- Creatinine Clearance < 30 mL/min
- Significant history neurologic, psychiatric, endocrinologic, metabolic, immunologic, or hepatic disease that would preclude participation in the study or compromise ability to give informed consent.
- Any history of other active malignancies within 3 years prior to study entry, with the exception of ade-quately treated in situ carcinoma of the cervix uterine, basal cell carcinoma of the skin or localized squa-mous cell carcinoma of the skin, previous malignancy confined and surgically resected with curative in-tent
- Subject who has malabsorption syndrome or other condition which precludes enteral route of administra-tion.
- Evidence of other clinically significant uncontrolled condition(s) including, but not limited to uncontrolled and/or active systemic infection (viral, bacterial or fungal)
- Active HBV positive hepatitis
- The following categories of patients HBV positive but with non evidence of active hepatitis may be con-sidered for the study:
- HBsAg positive with HBV DNA < 2000 UI/ml (inactive carriers); HBV DNA > 2000 UI/ml is criteria of ex-clusion
- HBsAg negative but HBsAb positive
- HBsAg negative but HBcAb positive
- Patients HBsAg positive with HBV DNA < 2000 UI/ml and HBsAg negative but HBcAb positive will be eligible for the study only if they accept to receive prophylactic Lamivudine 100 mg/daily for all the peri-od of treatment and at least for 12 months after the end of therapy. Treatment should be stopped in case of hepatitis reactivation.
- Active HCV positive hepatitis
- If female, the patient is pregnant or breast-feeding.

**Supplemental 2**

ABT-199 will be administered orally once daily according to the following ramp up:

• week 1 day 1: 20 mg

• week 1 day 2-3: 50 mg

• week 1 day 4-7: 100 mg

• week 2: 200 mg

• week 3; 400 mg

• week 4 and following: 800 mg
